# Nr4a1-dependent non-classical monocytes are important for macrophage-mediated wound healing in the large intestine

**DOI:** 10.3389/fimmu.2022.1040775

**Published:** 2023-01-18

**Authors:** Karin Heidbreder, Katrin Sommer, Maximilian Wiendl, Tanja M. Müller, Imke Atreya, Kai Hildner, Markus F. Neurath, Sebastian Zundler

**Affiliations:** ^1^ Department of Medicine 1, University Hospital Erlangen and Friedrich-Alexander-Universität Erlangen-Nürnberg, Erlangen, Germany; ^2^ Deutsches Zentrum Immuntherapie (DZI), University Hospital Erlangen, Erlangen, Germany

**Keywords:** *Nr4a1* (Nur77), monocytes, macrophages, intestinal wound healing, gut homing

## Abstract

**Introduction:**

Macrophages play an important role in intestinal wound healing. However, the trajectories from circulating monocytes to gut macrophages are incompletely understood.

**Methods:**

Taking advantage of mice depleted for non-classical monocytes due to deficiency for the transcription factor Nr4a1, we addressed the relevance of non-classical monocytes for large intestinal wound healing using flow cytometry, in vivo wound healing assays and immunofluorescence.

**Results:**

We show that wound healing in *Nr4a1*-deficient mice is substantially delayed and associated with reduced peri-lesional presence of macrophages with a wound healing phenotype.

**Discussion:**

Our data suggest that non-classical monocytes are biased towards wound healing macrophages. These insights might help to understand, how targeting monocyte recruitment to the intestine can be used to modulate intestinal macrophage functions.

## Background

The large surface of the gastrointestinal tract is constantly exposed to a plethora of exogenous substances and commensal as well as potentially pathogenic microbiota. Thus, to preserve the integrity of the host, tightly regulated programs are established to upkeep the mucosal barrier function (1, 2). This crucially involves sophisticated processes of intestinal wound healing, which are required to close defects in the epithelial and potentially also deeper layers of the mucosa in view of the constant challenges present in the lumen (3–5).

How important this is gets particularly evident in the context of pathology such as inflammatory bowel disease (IBD). Here, breaches in the mucosal barrier facilitate the translocation of antigens from a dysbiotic luminal microenvironment to the lamina propria, where inappropriate immune responses are evoked in genetically predisposed hosts (6). However, barrier defects are not only involved in these initial steps of the pathogenesis, but also key to approaches to resolve inflammation. Accordingly, numerous clinical trials have shown that mucosal healing is an important endpoint of therapeutic intervention (7–9) and predicts long-term remission (10).

This highlights the necessity to understand the molecular and cellular processes involved in intestinal wound healing (11, 12). Previous investigations have demonstrated an important role of different immune cells including macrophage subsets in promoting or counteracting mucosal repair (13, 14). In general, wound healing can be explained by a model involving several overlapping phases: Following initial hemostasis, there is an inflammatory phase marked by the recruitment of neutrophils and pro-inflammatory macrophages that fight bacteria challenging the wound area. In the further course, there is a transition into a proliferative phase, in which endothelial cells, fibroblasts and macrophages with a wound healing phenotype orchestrate neoangiogenesis and tissue restoration (15).

In this and other contexts, in addition to a subset of resident self-maintaining macrophages (16), the intestinal macrophage pool is constantly replenished by the recruitment of circulating monocytes differentiating into macrophages with different phenotypes (17). Classically, macrophages are categorized into M1 and M2 macrophages. While M1 macrophages are characterized by the production of pro-inflammatory cytokines such as TNF-α, IL-6 or IL-1β, M2 macrophages express molecules such as IL-10 and VEGF and have also been denoted as “wound healing macrophages” (18). Over the last decade, several reports have demonstrated that the reality is much more complex than this dichotomous view, since macrophage activation states are heterogeneous and context-sensitive (19).

There are also different subsets of circulating monocytes, in mice namely Ly6C^high^ classical monocytes considered as the main source of intestinal macrophages in homeostasis, and Ly6C^low^ non-classical monocytes patrolling along the vasculature (20). These non-classical monocytes are dependent on the transcription factor Nr4a1, which controls their bone marrow differentiation and survival (21). Of note, data from different tissues hint at a bias of non-classical monocytes towards differentiation into macrophages with a wound healing phenotype (22–24). However, this has not fully been established in the intestine and the impact of Nr4a1 for macrophage-mediated intestinal wound healing has not been addressed so far.

Here, we took advantage of *Nr4a1*-deficient mice and show that circulating non-classical monocytes and intestinal macrophages are disturbed in these mice, which is associated with delayed wound healing. Thus, our data add to the understanding of repair processes in the gut and might help to tailor future efforts to promote mucosal regeneration in the context of IBD.

## Methods

### Mice

*Nr4a1* ^−/−^ mice were obtained from The Jackson Laboratory (B6;129S2-*Nr4a1^tm1Jmi^*/J). C57BL/6 (WT) mice were purchased from Janvier Labs. All mice were housed in individually ventilated cages with a regular 12-hour day-night cycle and used for experiments according to approval by the Government of Lower Franconia in compliance with all relevant ethical regulations.

### Isolation of mouse cells

Bone marrow cells were isolated as previously described (24). Briefly, the femurs of age- and sex-matched *Nr4a1*-deficient and -proficient mice were dissected and flushed with a 27 3/4G needle through a 70 μm nylon strainer and washed two times with PBS.

To obtain splenocytes, freshly isolated spleens were mashed through a 40 µm cell strainer with the plunger of a 1 ml syringe. Subsequently, the cell pellet was resuspended in 3 ml of ammonium-chloride-potassium lysis buffer (155 mM ammonium chloride; 19 mM potassium hydrogen carbonate and 0.68 mM EDTA; pH 7.27) and gently shaken for 3 minutes to lyse erythrocytes.

Lamina propria mononuclear cells (LPMCs) were isolated from the colon using the Lamina Propria Dissociation Kit (Miltenyi Biotec) according to the manufacturer’s instructions followed by Percoll density gradient centrifugation (GE Healthcare).

Peripheral blood was taken from the facial vein. In order to remove erythrocytes, 2 ml of 1x BD Pharm Lyse™ lysing solution (BD Bioscience) was added to 100 µl of whole blood and incubated for 15 minutes at room temperature.

### Flow cytometry

To investigate the quantity and phenotype of monocytes, bone marrow cells, splenocytes, LPMCs and peripheral blood cells were analyzed by flow cytometry after staining for viable cells using the eBioscience Viability dye eFluor 780 (Invitrogen), blockade of unspecific binding with Fc Blocking Reagent (Miltenyi) and cell surface staining with the antibodies listed in **Supplementary Table 1**.

For the analysis of macrophages, splenocytes or LPMCs were treated and stained with the antibodies listed in **Supplementary Table 2** and **Supplementary Table 3**, respectively.

All samples were fixed in 300 µl BD cell fix (BD Bioscience) for 1 hour at 4°C or FluoroFix™ Buffer (Biolegend) for 1 hour at room temperature and analyses were performed on an LSR Fortessa (BD Bioscience) instrument. For data analysis we used FlowJo software (v10.8.1).

### *In vivo* wound healing model

*In vivo* wound healing was performed as previously described (24). *Nr4a1* ^-/-^ and WT mice were anesthetized with isoflurane for colonoscopy, which was performed with a rigid mini-endoscopy system (Karl Storz, SCB Xenon 175). On day 0, the intestinal mucosal wounds were inflicted with a biopsy forceps (Karl Storz 61029D with biopsy forceps 61071ZJ) in the descending colon. Afterwards, the wound diameters were documented by colonoscopy on day 0-7. The wound diameters were measured with Image J and related to the initial wound diameter on day 0.

### Immunofluorescence

Colon tissue from mice was obtained at day 5 after wounding as described above. A biopsy punch was used to retrieve wounds with peri-lesional tissue, which were immediately frozen in liquid nitrogen and embedded in OCT compound (Tissue Tek, Sakura). The embedded wounds were cut with Leica CM3050 S (layer thickness 10 µm). Cryosections were fixed with 4% PFA. Next, unspecific binding sites were blocked with Avidin/Biotin-Blocking-Kit (Vector Laboratories) and with protein blocking reagent (ROTI^®^ImmunoBlock, Roth) supplemented with 5% BSA (PAN-Biotech) and 20% goat serum (Vector Laboratories). Next, the slides were permeabilized with 0.1% triton X and were incubated with primary antibodies against Cd68 (polyclonal, abcam; dilution 1:200), Cd163 (TNKUPJ, Thermofisher; dilution 1:100), F4/80 (BM8, BioLegend; dilution 1:200), Cd206 (polyclonal, abcam; dilution 1:200), Arginase-1 (polyclonal, Novus Biologicals; dilution 1:300) or iNos (Polyclonal, abcam; dilution 1:50). Goat ant-rabbit AF488 (Invitrogen; dilution 1:200), goat anti-rat biotin followed by streptavidin-Cy3 (both BioLegend; dilution 1:200), goat anti-rat AF488 (abcam; dilution 1:200) and goat anti-rabbit Cy3 (Millipore; dilution 1:200) were used for detection. Nuclei were stained with Hoechst (Life Technologies). Analyses were performed with fluorescence microscopy (Leica DM6000B) and with confocal microscopy (Leica SP8).

To control for unspecific staining, a control panel was included for each slide. This control panel was only stained with the respective secondary antibodies but no primary antibody.

### Statistics

All statistical analyses were performed using the GraphPad Prism software (v9.0.2). All data sets were tested for normal distribution with the Shapiro-Wilk test in order to choose the appropriate parametric or non-parametric tests. When two groups were analyzed, an unpaired t-test was used for normally distributed data. For not normally distributed data, the Mann-Whitney test was chosen. If more than two groups were analyzed, a 2-way ANOVA was performed. Error bars in all graphs display the standard error of the mean (SEM). P values are indicated as follows: * p < 0.05, ** p < 0.01, *** p < 0.001, **** p < 0.0001.

## Results

### Peripheral blood non-classical monocytes and intestinal macrophages are reduced in *Nr4a1*-deficient mice

**Figure 1:**
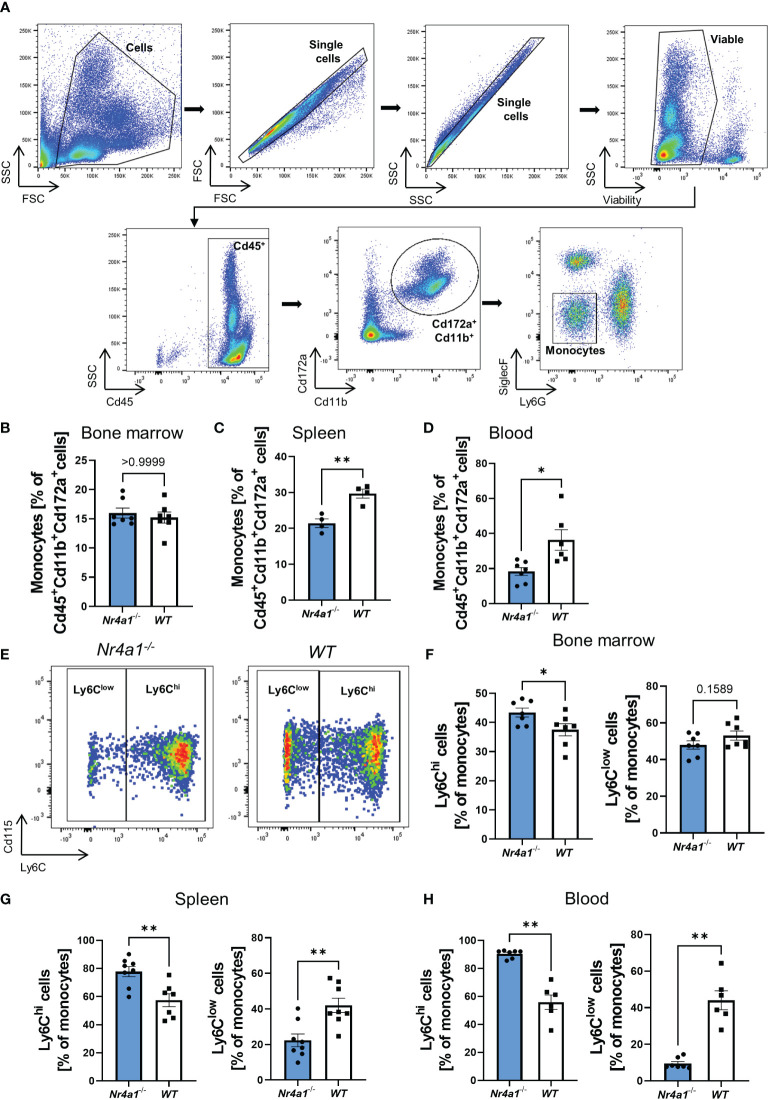
Depletion of non-classical monocytes in *Nr4a1^-/-^* mice. **(A)** Representative gating strategy for the identification of monocytes. Cells were gated based on forward and side scatter before selecting single cells and viable cells. Monocytes were defined as Cd45^+^Cd11b^+^Cd172a^+^Ly6G^-^SiglecF^-^. **(B–D)** Abundance of monocytes in the bone marrow **(B)**, spleen **(C)** and peripheral blood **(D)** gated as shown in **(A)**. **(E)** Representative gating for Ly6C^hi^ and Ly6C^low^ monocyte subsets in the peripheral blood of *Nr4a1^-/-^* and WT mice as % of monocytes. **(F–H)** Quantitative analysis of the different monocyte subsets in *Nr4a1^-/-^* and WT mice in the bone marrow (n=7) **(F)**, the spleen (n=8-9) **(G)** and the peripheral blood (n=6-7) **(H)**. * p < 0.05, ** p < 0.01.

In a first step, we aimed to confirm that *Nr4a1*-deficient mice display reduced numbers of non-classical monocytes. Thus, we isolated cells from the bone marrow, spleen and peripheral blood of *Nr4a1* ^-/-^ and WT mice and analyzed them by flow cytometry. Monocytes were defined as viable Cd45^+^Cd172a^+^Cd11b^+^Ly6G^-^SiglecF^-^ cells (25) (**Figure 1A**). While overall monocyte abundance in the bone marrow did not differ between *Nr4a1* ^-/-^ and WT mice, less monocytes were found in the spleens and the peripheral blood of *Nr4a1* ^-/-^ mice (**Figures 1B** **–D**). Importantly, the Cd45^+^Cd172a^+^Cd11b^+^ parent population remained stable, indicating a genuine effect on monocytes (**Supplementary Figures 1A–F**).

We further quantified monocyte subsets based on Ly6C expression defining non-classical monocytes as Ly6C^low^ and classical monocytes as Ly6C^hi^ (**Figure 1E**). In the bone marrow, classical monocytes were somewhat higher in *Nr4a1* ^-/-^ compared to WT mice and, accordingly, non-classical monocytes were slightly reduced (**Figure 1F** and **Supplementary Figure 1G**). In consistence with previous literature, these differences were substantially increased in the spleen and the peripheral blood (**Figures 1G, H**), clearly indicating extensive depletion of circulating non-classical monocytes in *Nr4a1*-deficient mice. A similar trend was also observed in the colon lamina propria (**Supplementary Figure 1H**).

**Figure 2:**
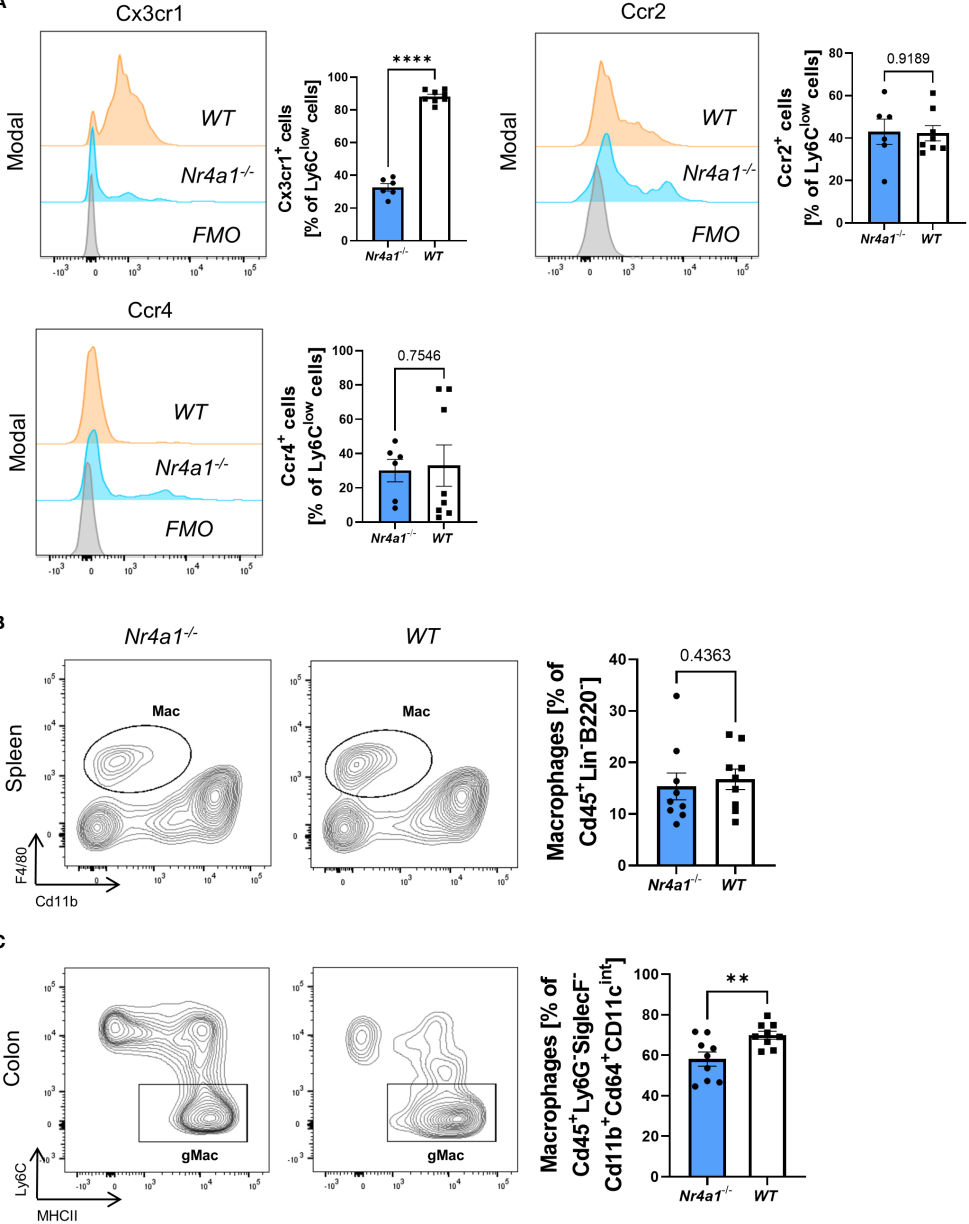
Alteration of Cx3cr1 expression on non-classical monocytes and reduction of intestinal macrophages in *Nr4a1*-deficient mice. **(A)** Representative histograms of the expression of the chemokine receptors Cx3cr1, Ccr2 and Ccr4 on Ly6C^low^ peripheral blood monocytes (left) and quantitative analysis (right) in *Nr4a1* ^-/-^ (n=6) and WT (n=8) mice from three independent experiments. **(B)** Representative (left) and quantitative flow cytometry (right) of splenic macrophages gated as described in **Supplementary Figure 2A** in *Nr4a1^-/-^* (n=9) and WT (n=9) mice. **(C)** Representative (left) and quantitative flow cytometry of viable Cd45^+^Ly6G^-^Cd11b^+^SiglecF^-^Cd64^+^Cd11c^int^MHCII^+^Ly6C^-^ macrophages in the colon lamina propria of *Nr4a1^-/-^* (n=9) and WT (n=9) mice. Three independent experiments, ** p < 0.01, **** p < 0.0001. FMO, Fluorescence Minus One Control.

Aiming to elucidate whether there are qualitative in addition to quantitative alterations in *Nr4a1*-deficient non-classical monocytes in the peripheral blood, we analyzed their expression of chemokine receptors. Interestingly, while the expression of Ccr2 and Ccr4 did not differ between *Nr4a1*-deficient and -proficient non-classical monocytes Cx3cr1 expression was clearly reduced (**Figure 2A**) suggesting that function of *Nr4a1*-deficient non-classical monocytes is altered in addition to abundance.

Since circulating monocytes are the pool for monocyte-derived macrophages in tissues such as the gut, we further investigated the abundance of macrophages in peripheral tissues. While we observed no reduction of Cd45^+^Lin^-^B220^-^F4/80^+^Cd11b^+^ macrophages in the spleens (25) of *Nr4a1*-deficient compared with *Nr4a1*-proficient mice (**Supplementary Figures 2A**, **Figure 2B**), the abundance of CD45^+^Ly6G^-^SiglecF^-^Cd11b^+^Cd64^+^Cd11c^int^MHCII^+^Ly6C^-^ macrophages in the large intestine (25) of *Nr4a1* ^-/-^ mice was significantly reduced, while the parent population remained stable (**Supplementary Figures 2B**, **Figure 2C**, **Supplementary Figure 3A**).

Collectively, these data showed that reduced circulating non-classical monocyte abundance in *Nr4a1*-deficient mice is associated with lower macrophage frequency in the gut.

### Experimental intestinal wound healing is delayed in *Nr4a1*-deficient mice

**Figure 3:**
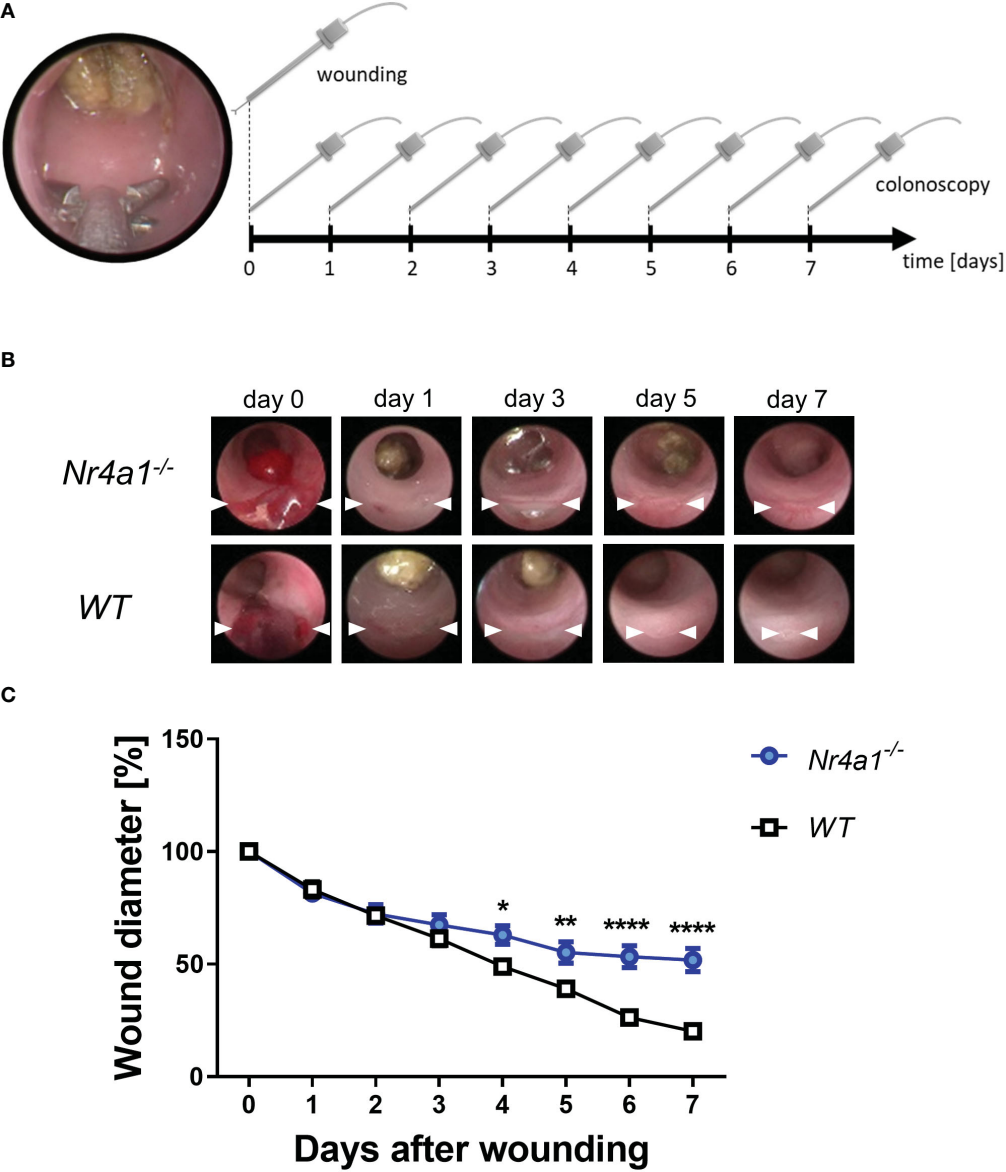
Delayed wound healing in the colon of *Nr4a1^-/-^* mice. **(A)** Schematic depiction of the *in vivo* wound healing model. The intestinal mucosal wounds were inflicted with a biopsy forceps (day 0) and subsequently followed up by endoscopy (day 0-7). **(B)** Representative colonoscopy images of large intestinal wounds in the descending colon at the indicated time points. **(C)** Quantitative analysis of relative wound diameters over time (n=8-9 per group). The indicated wound diameters were related to the initial wound diameter on day 0. * p < 0.05, ** p < 0.01, **** p < 0.0001.

To address the functional relevance of these observations in the context of intestinal wound healing, we took advantage of a well-established experimental model (24) using a biopsy forceps to induce mucosal wounds in *Nr4a1* ^-/-^ and WT mice (**Figure 3A**). The wounds of each animal were documented daily by colonoscopy (representative pictures in **Figure 3B**) and the wound diameters were measured and analyzed with ImageJ. Measurements were normalized to the initial wound diameter (day 0). These analyses demonstrated that wound repair was substantially delayed in *Nr4a1* ^-/-^ mice (**Figures 3B** **, C**). Interestingly, no differences were observed up to day 3 after wounding indicating that the early phase of wound healing is not affected by Nr4a1.

### Reduced peri-lesional presence of wound healing macrophages in *Nr4a1*-deficient mice

Earlier studies had suggested that delayed wound healing is associated with disturbances in the balance of macrophage subsets and in particular with a reduction of wound healing macrophages in the late phase of wound healing (24).

**Figure 4:**
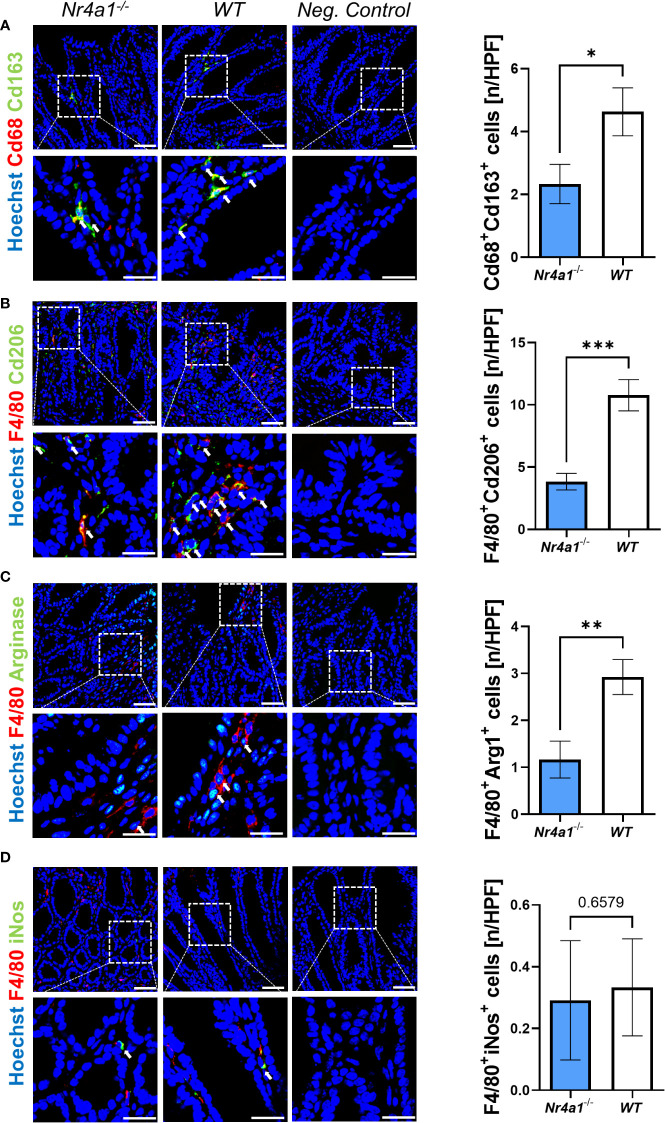
Reduced perilesional presence of wound healing macrophages in the colonic mucosa of *Nr4a1^-/-^* mice compared to WT mice. Representative (left) and quantitative immunofluorescence (right) for the perilesional co-expression of Cd68 and Cd163 **(A)**, F4/80 and Cd206 **(B)**, F4/80 and Arg1 **(C)** and F4/80 and inducible nitric oxide synthase (iNos) **(D)** in intestinal wound areas of *Nr4a1^-/-^* mice (n=8) and WT mice (n=9) on day 5 after injury. Scale bars 50 µm (upper panels), 25 µm (lower panels); HPF, high-power field. * p<0.05, ** p<0.01, *** p<0.001.

Thus, we finally explored the presence of macrophage subsets in the wound bed of intestinal wounds at day 5 after wounding by immunofluorescence. We found significantly less cells co-expressing the pan-macrophage marker Cd68 and Cd163, a scavenger receptor preferentially expressed on wound healing macrophages, in the wound area in *Nr4a1* ^-/-^ mice compared with WT mice (**Figure 4A**). The same was the case, when staining for the combination of the pan-macrophage marker F4/80 and Cd206 (**Figure 4B**), a C-type lectin that is also preferentially expressed on wound healing macrophages (26–28). Moreover, a similar pattern was observed upon staining with F4/80 in combination with Arginase-1 (**Figure 4C**), another marker of wound healing macrophages (29, 30). To the contrary, no differences were noted for F4/80^+^iNos^+^ cells (**Figure 4D**) with an M1-like pro-inflammatory phenotype (31, 32). Interestingly, the analysis of the pan macrophage markers Cd68 and F4/80 showed no significant difference in the wound area in *Nr4a1* ^-/-^ mice compared to WT mice (**Supplementary Figure 3B**) indicating that the effects observed are subset-specific.

Taken together, these data demonstrated that wound healing macrophages, but not pro-inflammatory macrophages are reduced during intestinal wound repair in *Nr4a1*-deficient mice.

## Discussion

Monocyte-derived macrophages are centrally involved in processes of tissue regeneration (33). They appear in heterogeneous activation states, which serve different functions during wound healing: While the early phase is marked by the presence of macrophages with pro-inflammatory phenotypes, tissue repair and resolving macrophages are observed later on (34–36).

Although self-renewing local macrophages have been described in the gut (16), a relevant part of the intestinal macrophages is constantly replenished by monocytes homing to the gut from the circulation (17). In analogy to the predominantly T cell-focused therapeutic approaches aiming at reducing inflammation in IBD by preventing gut homing *via* the integrin α4β7 (37, 38), this fact might provide opportunities to modulate macrophage-dependent processes such as mucosal healing in the intestine. However, circulating monocytes are also heterogeneous and targeted strategies therefore require to understand the developmental trajectories from specific monocyte subsets to tissue macrophages.

Thus, we aimed to determine the function and presence of macrophage subsets in mice deficient for the transcription factor Nr4a1 leading to specific depletion of non-classical monocytes (21). We show that this depletion is associated with delayed wound healing and reduced peri-lesional wound healing macrophage abundance. Thus, although it has to be underscored that this must not be understood as a causal proof of such trajectories, our data suggest that non-classical monocytes are important precursors of wound healing macrophages in the intestine.

This is well in line with previous literature. Auffray et al. had shown that a transcriptional signature resembling M2 macrophages is initiated in non-classical “patrolling” monocytes after recruitment to wound areas (22). Consistently, Olingy and colleagues demonstrated in a soft tissue injury model that non-classical monocytes are skewed towards wound healing macrophages (23) and, similarly, we had earlier suggested this for the gut based on latex bead uptake studies (24).

Thus, collectively, these data indicate that specifically targeting the gut homing of distinct monocyte subsets might be useful to modulate specific intestinal macrophage functions. This might indeed be feasible, since several lines of evidence point towards differential tissue homing pathways employed by classical and non-classical monocytes. E.g., it has been shown in atherosclerosis that the expression and function of chemokine receptors such as Ccr2, Ccr5 and Cx3cr1 differs between classical and non-classical monocytes (39). Similarly, P-selectin glycoprotein ligand 1 was specifically expressed on classical monocytes and determined their homing to atherosclerotic plaques in mice (40).

Our data are also in line with previously reported findings on the role of Nr4a1 for monocytes and macrophage differentiation. While non-classical monocytes seem to differentiate from classical monocytes (41), Nr4a1 has been demonstrated to be essential for this conversion (21, 42). Thus, our finding of reduced non-classical and increased classical monocytes in Nr4a1-deficient mice is probably reflecting differentiation block. On a functional level and consistent with our data, Honda et al. showed in a thermal injury model in the gut that Nr4a1-dependent Cx3cr1^+^Ccr2^low^ monocytes/macrophages were essential for tissue repair in the intestine (43). Conversely, Menezes and colleagues demonstrated that Ly6C^+^ monocytes that are different from Nr4a1-dependent non-classical Ly6C^low^ monocytes preferentially differentiate into iNos^+^ pro-inflammatory macrophages or monocyte-derived dendritic cells (44). However, other data also underscore that tissue-specific mechanisms seem to exist, since in the myocardium, Ly6C^low^ monocytes did not differentiate into macrophages, whereas Ly6C^hi^ monocytes gave rise both to pro-inflammatory and, in an Nr4a1-dependent mechanism, to reparative macrophages (45).

A limitation that needs to be mentioned is that, although we defined non-classical and classical monocytes according to well established protocols available in the literature (25), which comprise the exclusion of other myeloid cell subset such as neutrophils (based on Ly6G expression), eosinophils (based on Siglec F expression), NK cells (based on Cd172a and Cd11b expression) and cDC1 cells (based on Cd172a expression), we cannot formally exclude a contamination of our population by Cd172^+^ cDC2 cells. It will be important to further address this possibility in future studies.

In conclusion, our data further substantiate the concept of preferential developmental pathways between circulating monocyte and intestinal macrophage subsets and suggest that these are of functional relevance in intestinal wound healing. These insights might help to design future therapeutic approaches interfering with intestinal macrophage-dependent processes based on modulating the recruitment of monocytes to the gut.

## Data availability statement

The original contributions presented in the study are included in the article/**Supplementary Material**. Further inquiries can be directed to the corresponding author.

## Ethics statement

The animal study was reviewed and approved by Regierung von Unterfranken.

## Author contributions

KHe and KS performed the experiments. KHe, KS, MN, and SZ designed the research. MW, TM, IA, KHi, MN, and SZ contributed samples or protocols. KHe, KS, MW, MN, and SZ analyzed and interpreted the data. KHe and SZ drafted the manuscript; all authors critically read and revised the manuscript for important intellectual content and approved the final version.

## Supplementary material

Supplementary Figure 1
**(A–C)** Quantitative analyses of the Cd45^+^Cd172a^+^Cd11b^+^ parent population of the bone marrow **(A)**, spleen **(B)** and peripheral blood **(C)** in *Nr4a1* ^-/-^ (n=7-8) and WT (n=7-9) mice underlying **Figure 1**. **(D–F)** Quantitative flow cytometry analyses of SiglecF^+^Ly6G^-^ (left) and SiglecF^-^Ly6G^+^ cells (right) in the bone marrow **(D)**, spleen **(E)** and peripheral blood **(F)** (n=6-9). **(G)** Representative gating for the identification of Ly6C^hi^ and Ly6C^low^ monocyte subsets in the bone marrow of *Nr4a1* ^-/-^ and WT mice **(H)** Quantitative analysis of Ly6C^hi^ and Ly6C^low^ monocytes in the lamina propria of *Nr4a1* ^-/-^ (n=7) and WT mice (n=6). * p < 0.05.

Supplementary Figure 2
**(A)** Representative gating strategy for the identification of splenic macrophages. Linage markers included Cd49b, Cd3, Cd19 and Ly6G. **(B)** Representative gating strategy for the identification of gut macrophages.

Supplementary Figure 3
**(A)** Representative (left) and quantitative flow cytometry (right) of the expression of Cd64^+^Cd11c^int^ cells in LPMCs of *Nr4a1* ^-/-^ (n=9) and WT (n=9) mice. **(B)** Quantitative immunofluorescence for the perilesional expression of Cd68 and F4/80 for the Arg1 staining in intestinal wound areas of *Nr4a1^-/-^* mice (n=9) and WT mice (n=9) on day 5 after injury.
