# Supplementary figures and images for: Nr4a1-dependent non-classical monocytes are important for macrophage-mediated wound healing in the large intestine
Source: Front Immunol. 2023 Jan 18;13:1040775. doi: 10.3389/fimmu.2022.1040775 (PMC9890957; doi:10.3389/fimmu.2022.1040775)

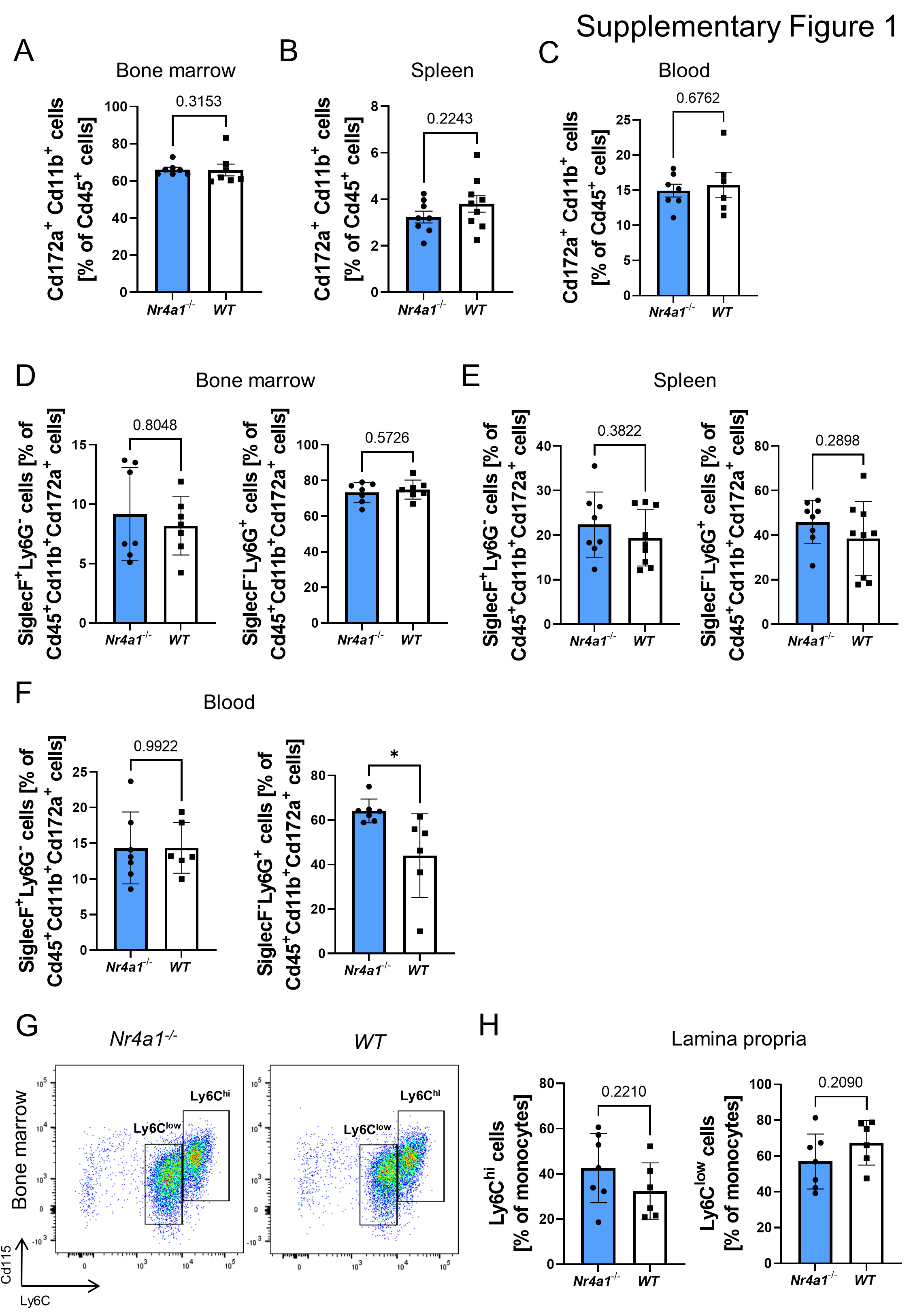

Supplement: Supplementary Figure 1 — (A–C) Quantitative analyses of the Cd45+Cd172a+Cd11b+ parent population of the bone marrow (A), spleen (B) and peripheral blood (C) in Nr4a1 -/- (n=7-8) and WT (n=7-9) mice underlying Figure 1 . (D–F) Quantitative flow cytometry analyses of SiglecF+Ly6G- (left) and SiglecF-Ly6G+ cells (right) in the bone marrow (D), spleen (E) and peripheral blood (F) (n=6-9). (G) Representative gating for the identification of Ly6Chi and Ly6Clow monocyte subsets in the bone marrow of Nr4a1 -/- and WT mice (H) Quantitative analysis of Ly6Chi and Ly6Clow monocytes in the lamina propria of Nr4a1 -/- (n=7) and WT mice (n=6). * p < 0.05. [file Image_1.tif]

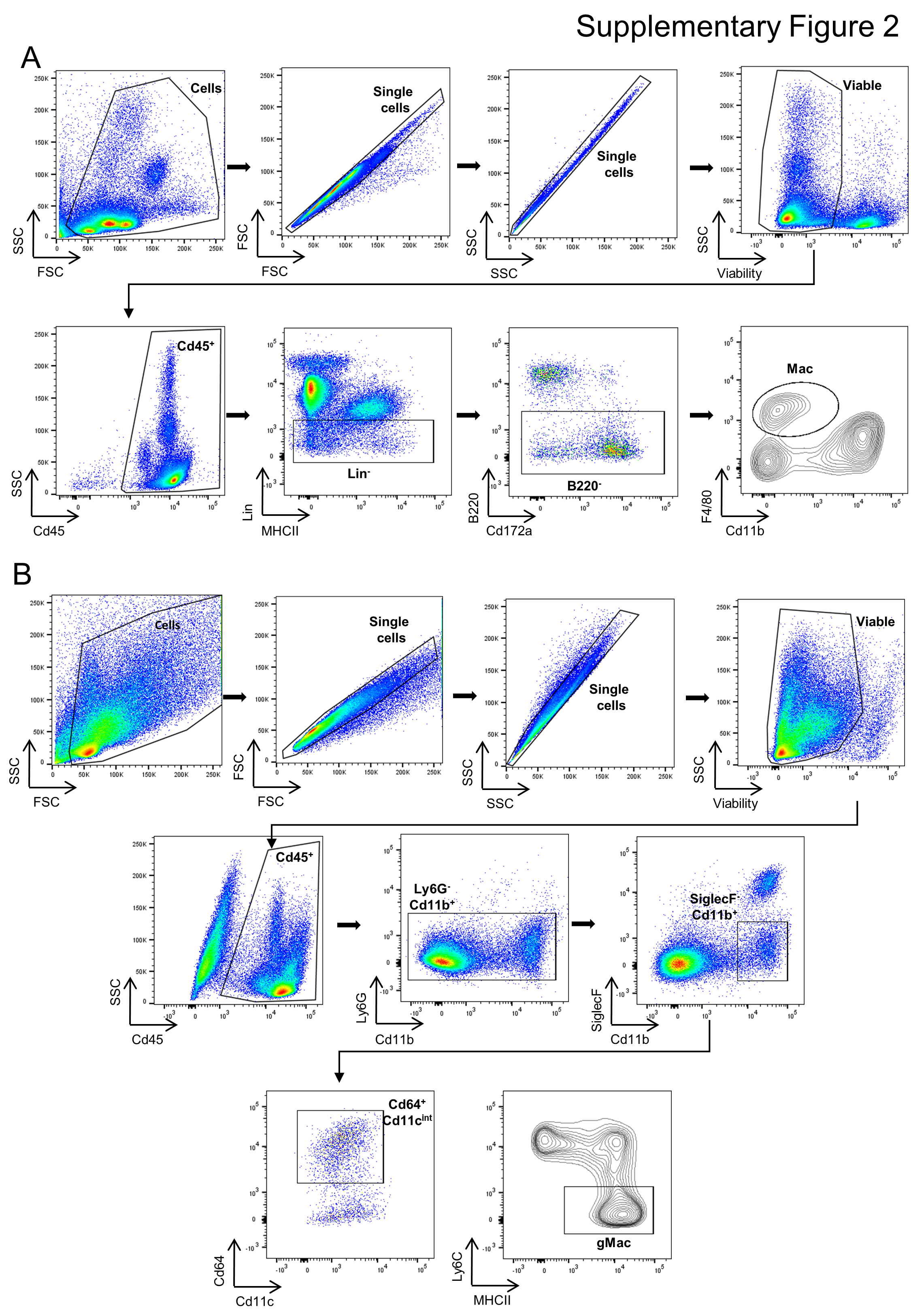

Supplement: Supplementary Figure 2 — (A) Representative gating strategy for the identification of splenic macrophages. Linage markers included Cd49b, Cd3, Cd19 and Ly6G. (B) Representative gating strategy for the identification of gut macrophages. [file Image_2.tif]

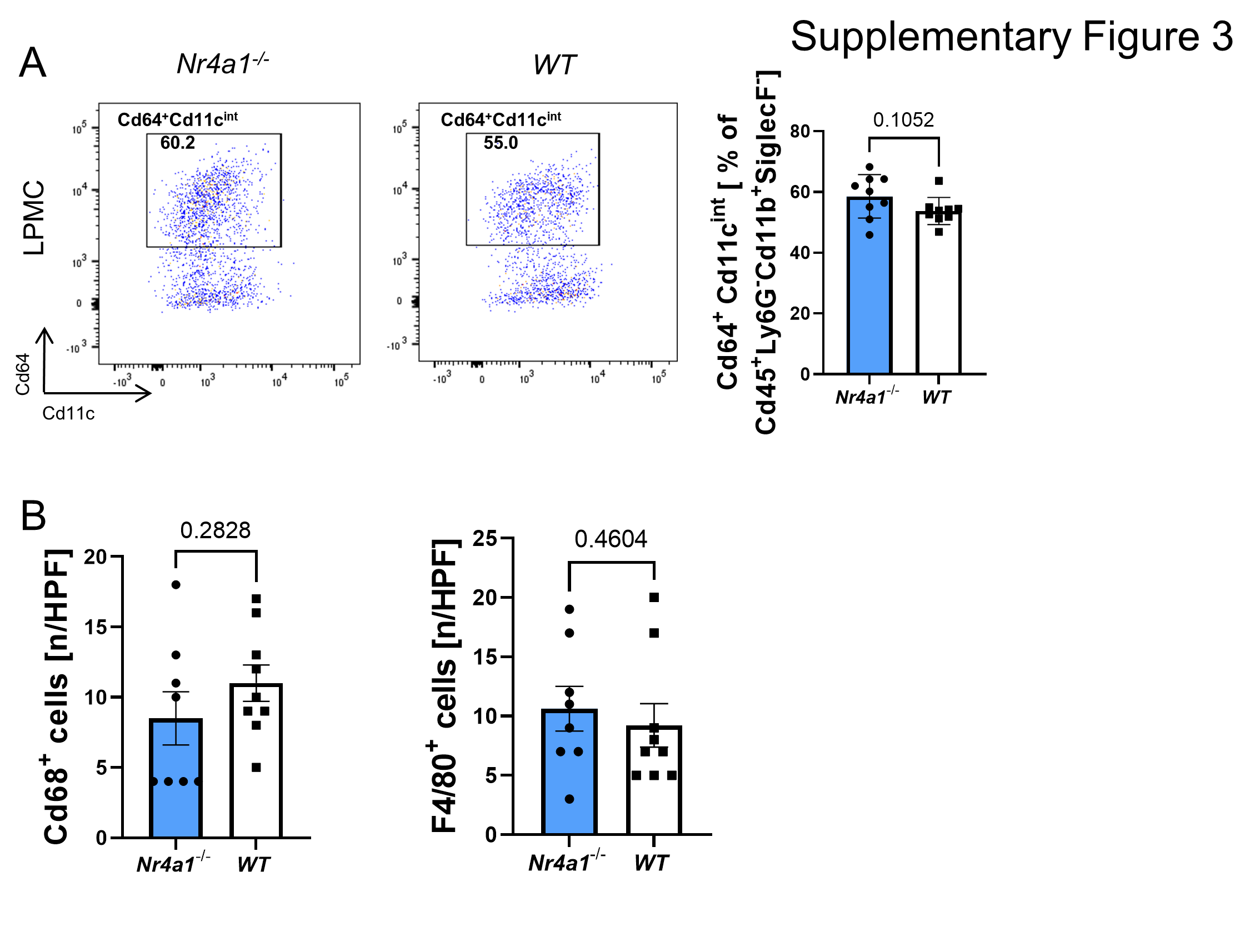

Supplement: Supplementary Figure 3 — (A) Representative (left) and quantitative flow cytometry (right) of the expression of Cd64+Cd11cint cells in LPMCs of Nr4a1 -/- (n=9) and WT (n=9) mice. (B) Quantitative immunofluorescence for the perilesional expression of Cd68 and F4/80 for the Arg1 staining in intestinal wound areas of Nr4a1-/- mice (n=9) and WT mice (n=9) on day 5 after injury. [file Image_3.tif]
