# Supplementary material for: Nr4a1-dependent non-classical monocytes are important for macrophage-mediated wound healing in the large intestine
Source: Front Immunol. 2023 Jan 18;13:1040775. doi: 10.3389/fimmu.2022.1040775 (PMC9890957; doi:10.3389/fimmu.2022.1040775)
Supplement: Supplementary file 4 [file Table_1.pdf]

Supplementary Table 1: List of antibodies for the staining of monocytes in bone marrow, spleen, LPMC and blood

Target	Fluorochrome	Clone	Manufacturer	Dilution factor
Cd45	BUV395	30-F11	BD Bioscience	1:100
Ly6G	BV711	1A8	Biolegend	1:200
SiglecF	BV421	E50-2440	BD Bioscience	1:200
Cd11b	BV650	M1/70	Biolegend	1:200
Cd172a	PE/Cy7	P84	Biolegend	1:80
Cd115	BV605	AF598	Biolegend	1:50
Ly6C	FITC	HK1.4	Biolegend	1:200
Ccr2	BV510	SA203G11	Biolegend	1:100
Ccr4	PE	2G12	Biolegend	1:100
Cx3cr1	APC	QA16A03	Biolegend	1:100

Supplementary Table 2: List of antibodies for the staining of splenic macrophages

Target	Fluorochrome	Clone	Manufacturer	Dilution factor
Cd45	BUV395	30-F11	BD Bioscience	1:100
MHCII	AF700	M5/114.15.2	eBioscience	1:200
Cd11b	APC	M1/70	Biolegend	1:100
Cd172a	PE/Cy7	P84	Biolegend	1:80
Cd45R (B220)	Vio Blue	RA3-6B2	Miltenyi	1:40
Cd49b	FITC	HMα2	Biolegend	1:200
Cd3	FITC	17A2	Biolegend	1:50
Cd19	FITC	6D5	Biolegend	1:100

Ly6G	FITC	1A8	Biolegend	1:200
F4/80	PerCP/Cy5.5	BM8	Biolegend	1:100

Supplementary Table 3: List of antibodies for the staining of lamina propria macrophages

Target	Fluorochrome	Clone	Manufacturer	Dilution factor
Cd45	BUV395	30-F11	BD Bioscience	1:100
Ly6G	BV711	1A8	Biolegend	1:50
SiglecF	BV421	E50-2440	BD Bioscience	1:50
Cd11b	BV650	M1/70	Biolegend	1:50
Cd64 (FcγRI)	PE	X54-5/7.1	Biolegend	1:100
Cd11c	BV510	N418	Biolegend	1:50
MHCII	AF700	M5/114.15.2	eBioscience	1:50
Ly6C	FITC	HK1.4	Biolegend	1:50
